# Hospitalization and survival of solid organ transplant recipients with coronavirus disease 2019: A propensity matched cohort study

**DOI:** 10.1371/journal.pone.0278781

**Published:** 2022-12-19

**Authors:** Joshua T. Swan, Elsie Rizk, Stephen L. Jones, Nwabunie Nwana, Juan C. Nicolas, Anh Thu Tran, Jiaqiong Xu, Tariq Nisar, Terri Menser, Stephanie G. Yi, Linda W. Moore, Howard J. Huang, R. Mark Ghobrial, A. Osama Gaber, Richard J. Knight

**Affiliations:** 1 Department of Pharmacy, Houston Methodist, Houston, TX, United States of America; 2 Department of Surgery, Houston Methodist, Houston, TX, United States of America; 3 Center for Outcomes Research, Houston Methodist, Houston, TX, United States of America; 4 Department of Internal Medicine, Houston Methodist, Houston, TX, United States of America; Faculty of Medicine, Saint-Joseph University, LEBANON

## Abstract

**Background:**

Solid organ transplant (SOT) recipients are predicted to have worse COVID-19 outcomes due to their compromised immunity. However, this association remains uncertain because published studies have had small sample sizes and variability in chronic comorbidity adjustment.

**Methods:**

In this retrospective cohort study conducted at a multihospital health system, we compared COVID-19 outcomes and survival up to 60 days following hospital admission in SOT recipients taking baseline immunosuppressants versus hospitalized control patients.

**Results:**

The study included 4,562 patients who were hospitalized with COVID-19 (108 SOT recipients and 4,454 controls) from 03/2020 to 08/2020. Mortality at 60 days was higher for SOT recipients (17% SOT vs 10% control; unadjusted odds ratio (OR) = 1.74, 95% confidence interval (CI) 1.04–2.91, P = 0.04). We then conducted a 1:5 propensity matched cohort analysis (100 SOT recipients; 500 controls) using age, sex, race, body mass index, hypertension, diabetes, chronic kidney disease, liver disease, admission month, and area deprivation index. Within 28 days of admission, SOT recipients had fewer hospital-free days (median; 17 SOT vs 21 control; OR = 0.64, 95%CI 0.46–0.90, P = 0.01) but had similar ICU-free days (OR = 1.20, 95%CI 0.72–2.00, P = 0.49) and ventilator-free days (OR = 0.91, 95%CI 0.53–1.57, P = 0.75). There was no statistically significant difference in 28-day mortality (9% SOT vs 12% control; OR = 0.76, 95%CI 0.36–1.57, P = 0.46) or 60-day mortality (16% SOT vs 14% control; OR = 1.15, 95%CI 0.64–2.08, P = 0.64).

**Conclusions:**

Hospitalized SOT recipients appear to need additional days of hospital care but can achieve short-term mortality outcomes from COVID-19 that are similar to non-SOT recipients in a propensity matched cohort study.

## 1. Background

In March 2020, the World Health Organization (WHO) declared coronavirus disease 2019 (i.e., SARS-CoV-2 virus; COVID-19) a global pandemic [1]. To date, more than 240 million cases have been confirmed with more than 4.9 million reported deaths [2]. Surges in COVID-19 cases overwhelm hospitals and are associated with inferior health outcomes [3]. To guide health policy, the healthcare community seeks to identify patients at increased risk for deleterious COVID-19 outcomes.

Immunosuppressed patients may experience increased risk for contracting the SARS-CoV-2 virus and suffering worse clinical outcomes compared to the general population. Solid organ transplant (SOT) recipients are of particular interest given their high healthcare utilization, compromised immunity, and fragile organ allografts. Due to these concerns, transplant centers across the United States suspended live donor transplantation and restricted deceased donor transplantation during COVID-19 surges [4]. Several cases series published early in the pandemic described outcomes among SOT recipients. Yi et al. reported that among 21 SOT recipients diagnosed with COVID-19 at our institution from January to April 2020, 14 (67%) required hospitalization, and 7 (33%) required admission to the intensive care unit (ICU) [5]. Fung et al. reported that among 10 SOT recipients with COVID-19, 7 (70%) were hospitalized and 3 (30%) required admission to the ICU [6]. Akalin et al. reported death in 10 (28%) of 36 kidney transplant recipients within 21 days of COVID-19 diagnosis [7].

Although these reports suggested SOT might be associated with worse COVID-19 outcomes, these case series describe small samples of SOT recipients without comparison to meaningful control groups. Although immunocompromised patients were expected to have worse COVID-19 outcomes, this association has not been clearly demonstrated among SOT recipients in published studies due to sample size limitations, short or variable observation periods, inclusion of some SOT recipients who were not taking immunosuppression at baseline, and variability in adjustment for chronic comorbidities [8–13]. Therefore, this study compared clinical outcomes following hospital admission for COVID-19 management for a large cohort of patients with vs without a history of SOT. Propensity score matching adjusted for imbalances in comorbidities and socio-economic status, measured using area deprivation index (ADI) which is associated with increased risk of COVID-19 infection and associated mortality [14–16]. Healthcare utilization was evaluated for 28 days, and death was evaluated for 60 days to provide additional follow-up.

## 2. Materials and methods

### 2.1. Study design and setting

The multihospital, retrospective cohort study was conducted at the Houston Methodist health system and was approved by the Houston Methodist Research Institute’s Institutional Review Board with a waiver of informed consent (PRO00027760).

### 2.2. Patients

The study included all hospitalized adults who were diagnosed with COVID-19 by reverse-transcriptase polymerase chain reaction (PCR) between March 17, 2020 to August 24, 2020 at any healthcare facility in our health system and were included in our health system’s COVID-19 Surveillance and Outcomes Registry (CURATOR) [17]. Index hospital admission was defined as the first hospital admission where COVID-19 was diagnosed or treated. Patients who had emergency department (ED) visits with an "inpatient" status in the medical record were included even if discharged from the ED to account for inability to transfer to a hospital unit due to limited bed availability during surges of the pandemic. Otherwise included patients were categorized into those with a history of solid organ transplant (SOT group) versus those without (control group). Patients with a first positive PCR for COVID-19 detected 7 days after the index hospital admission where excluded to remove potential nosocomial cases of COVID-19. Patients with a first positive PCR for COVID-19 detected more than 7 days before the index hospital admission were excluded to remove patients with a recent history of COVID-19 who were being hospitalized for other conditions. Pregnant patients were excluded to remove childbirth encounters among asymptomatic SARS-CoV-2 positive mothers. Patients who were missing data for any variables used to construct a propensity score were excluded. Patients with a history of SOT who experienced chronic allograft failure and were no longer taking immunosuppressant therapy immediately prior to the COVID-19 diagnosis were excluded from the study.

### 2.3. Outcomes

Study outcomes evaluated healthcare resource utilization within 28 days of index hospital admission and all-cause mortality within 28 and 60 days of index hospital admission [18, 19]. We expected a high burden of mortality, which is a competing event that confounds interpretation of hospital length of stay and healthcare resource utilization. Therefore, we constructed three variables that evaluate healthcare resource utilization that accounted for death: days alive and free from the hospital (hospital free days; HFDs), days alive and free from the ICU (ICU free days; IFDs), and days alive and free from mechanical ventilation (ventilator free days; VFDs). Example calculations of HFDs are shown in S1 Table. Days in long-term care facilities or skilled nursing facilities were counted as hospital days for calculation of HFDs. Days in the ED, procedural areas, and intermediate care units (IMUs) were not counted as ICU days for calculation of IFDs.

### 2.4. Statistical analysis

This study tested the hypothesis that among patients hospitalized for COVID-19, patients with a history of SOT have fewer HFDs over a 28-day period (increased healthcare utilization) compared to patients without a history of SOT. For the unadjusted analysis, all outcomes were evaluated in the full cohort of patients who met inclusion and exclusion criteria. This study also used propensity score matched analysis to remove potential confounding by indication. A propensity score was calculated using age, sex, race, body mass index (BMI), hypertension, diabetes mellitus, chronic kidney disease (CKD), liver disease, admission month, and ADI. Patients in the SOT group were matched with patients from the control group using 1:5 nearest neighbor matching without replacement. The balance of covariates used to construct the propensity score were evaluated before and after matching. An absolute standardized difference ≤0.1 indicated balance. Since all covariates were balanced after matching, covariates were not added to regression models for primary or secondary analyses. The primary analysis evaluated HFDs using a proportional odds model that accounted for matching to calculate a common odds ratio. An odds ratio less than 1 indicated increased healthcare utilization for patients in the SOT group. The same analytic approach was used for VFDs and IFDs. The outcome of 60-day mortality was analyzed using conditional logistic regression that accounted for matching. As a sensitivity analysis, the proportional hazards assumption was evaluated for analyses of HFDs, IFDs, and VFDs. If needed, outcomes were collapsed to 2-day increments to satisfy the proportional hazards assumption. A second sensitivity analysis evaluated HFDs, VFDs, and IFDs as continuously scaled outcomes using generalized estimating equations that assumed a gaussian distribution and accounted for matching. A third sensitivity analysis evaluated and adjusted for imbalance of critical illness on admission, which was defined as need for mechanical ventilation or ICU admission on hospital days 1 or 2. Subgroup analyses were conducted to stratify outcomes based on year of most recent transplant (2019–2020 versus prior to 2019) and organ type. A two-sided alpha of 0.05 was used to designate statistical significance. Statistical analyses and data management were conducted using STATA version 16 (StataCorp LP, College Station, Texas).

## 3. Results

### 3.1. Full cohort

A total of 4,562 patients met inclusion and exclusion criteria and were included in the full cohort (SOT group = 108 vs control group = 4,454; S1 Fig). Among 108 patients in the SOT group in the full cohort, 59% had kidney transplant, 87% were taking tacrolimus at baseline, 83% were taking prednisone at baseline, and 76% were taking mycophenolate mofetil (MMF) at baseline (Table 1). As part of institutional COVID-19 management, MMF was held or reduced in 93% (76 of 82) of SOT recipients who were taking the drug at baseline.

**Table 1:** Characteristics and immunosuppression therapy for SOT recipients.

Characteristics, *n* (%)	SOT (n = 108)
Year of last transplant	
2020	10 (9%)
2019	14 (13%)
Before 2019	83 (77%)
Unknown	1 (1%)
All transplanted organs	
Kidney	64 (59%)
Liver	13 (12%)
Lung	12 (11%)
Heart	5 (5%)
Multiorgan transplant ^†^	14 (13%)
Cell-depleting antibodies administered within 90 days before diagnosis ^‡^	4 (4%)
Antithymocyte globulin	2 (2%)
Rituximab	2 (2%)
Bortezomib	1 (1%)
Carfilzomib	1 (1%)
Immunosuppressants active at the time of diagnosis ^‡^	
Tacrolimus	94 (87%)
Cyclosporine	7 (7%)
Mycophenolate mofetil	82 (76%)
Azathioprine	4 (4%)
Sirolimus	9 (8%)
Everolimus	3 (3%)
Belatacept	4 (4%)
Prednisone	90 (83%)
Immunosuppressant modifications after COVID-19 diagnosis ^‡^	
Tacrolimus reduced or held	19 of 94 (20%)
Cyclosporine reduced or held	1 of 7 (14%)
Mycophenolate mofetil reduced or held	76 of 82 (93%)
Azathioprine reduced or held	2 of 4 (50%)
Sirolimus held	5 of 9 (56%)
Belatacept held	4 of 4 (100%)
Prednisone/Hydrocortisone replaced with another steroid ^§^	49 of 90 (54%)
Continue the same immunosuppressant regimen	15 of 108 (14%)

SOT, solid organ transplant

^†^ Included kidney/pancreas (*n* = 9), liver/kidney (*n* = 3), heart/liver (*n* = 1), and heart/kidney (*n* = 1);

^‡^ Non-mutually exclusive;

^§^ Prednisone was replaced with dexamethasone (*n* = 39), methylprednisolone (*n* = 3), hydrocortisone (*n* = 3), dexamethasone then methylprednisolone (*n* = 3), and dexamethasone followed by methylprednisolone and hydrocortisone (*n* = 1)

Using unadjusted analysis of the full cohort, median HFDs were 17 for the SOT group and 22 for the control group (odds ratio (OR) = 0.50, 95% confidence interval (CI) 0.36 to 0.69, P<0.001), median IFDs were 28 for the SOT group and 28 for the control group (OR = 0.92, 95%CI 0.60 to 1.41, P = 0.69), and median VFDs were 28 for the SOT group and 28 for the control group (OR = 0.75, 95%CI 0.47 to 1.20, P = 0.23). The incidence of death at 28 days was 10% for the SOT group and 8% for the control group (OR = 1.29, 95%CI 0.68 to 2.42, P = 0.44). The incidence of death at 60 days was 17% for the SOT group and 10% for the control group (OR = 1.74, 95%CI 1.04 to 2.91, P = 0.04).

### 3.2. Propensity matched cohort

The propensity score matched cohort included 600 patients (SOT group = 100 vs control group = 500; S1 Fig). Covariates were unbalanced prior to matching. All covariates were balanced after matching (Table 2). Compared to the control group in the full cohort prior to matching, the control group in the matched cohort appeared to have higher prevalence of CKD (58% vs 22%), hypertension (80% vs 63%), and diabetes (76% vs 47%).

**Table 2:** Balance of covariates before and after matching.

	Full cohort			Matched cohort		
	SOT	Controls	STD diff	SOT	Controls	STD diff
	(n = 108)	(n = 4,454)		(n = 100)	(n = 500)	
Male gender, n (%)	61 (56%)	2,286 (51%)	0.10	58 (58%)	283 (57%)	0.03
BMI, mean ± SD	29 ± 6	32 ± 8	0.39	29 ± 6	29 ± 7	0.05
CKD, n (%)	68 (63%)	992 (22%)	0.90	60 (60%)	288 (58%)	0.05
Liver disease, n (%)	5 (5%)	283 (6%)	0.08	5 (5%)	25 (5%)	0.00
Hypertension, n (%)	87 (81%)	2,827 (63%)	0.39	80 (80%)	400 (80%)	0.00
Diabetes mellitus, n (%)	85 (79%)	2,097 (47%)	0.69	78 (78%)	380 (76%)	0.05
Race			0.21			0.02
White	73 (68%)	2,753 (62%)		67 (67%)	331 (66%)	
Black	27 (25%)	1,145 (26%)		25 (25%)	129 (26%)	
Asian	5 (5%)	236 (5%)		5 (5%)	26 (5%)	
Other	3 (3%)	320 (7%)		3 (3%)	14 (3%)	
Age, mean ± SD	56 ± 13	59 ± 17	0.23	57 ± 13	58 ± 16	0.08
ADI, mean ± SD	60 ± 25	54 ± 26	-0.23	60 ± 25	58 ± 26	-0.06
Admission month, n (%)			0.27			0.05
March 2020	2 (2%)	103 (2%)		2 (2%)	9 (2%)	
April 2020	9 (8%)	365 (8%)		7 (7%)	35 (7%)	
May 2020	2 (2%)	278 (6%)		2 (2%)	12 (2%)	
June 2020	29 (27%)	1,184 (27%)		27 (27%)	139 (28%)	
July 2020	56 (52%)	1,956 (44%)		52 (52%)	260 (52%)	
August 2020	10 (9%)	568 (13%)		10 (10%)	45 (9%)	

ADI, area deprivation index; BMI, body mass index; CKD, chronic kidney disease; SD, standard deviation; SOT, solid organ transplant; STD diff, standardized difference

An absolute STD diff <0.1 indicated that the covariate was balanced between study groups. All covariates were balanced after matching.

### 3.3. Primary outcome: HFDs

**Fig 1:**
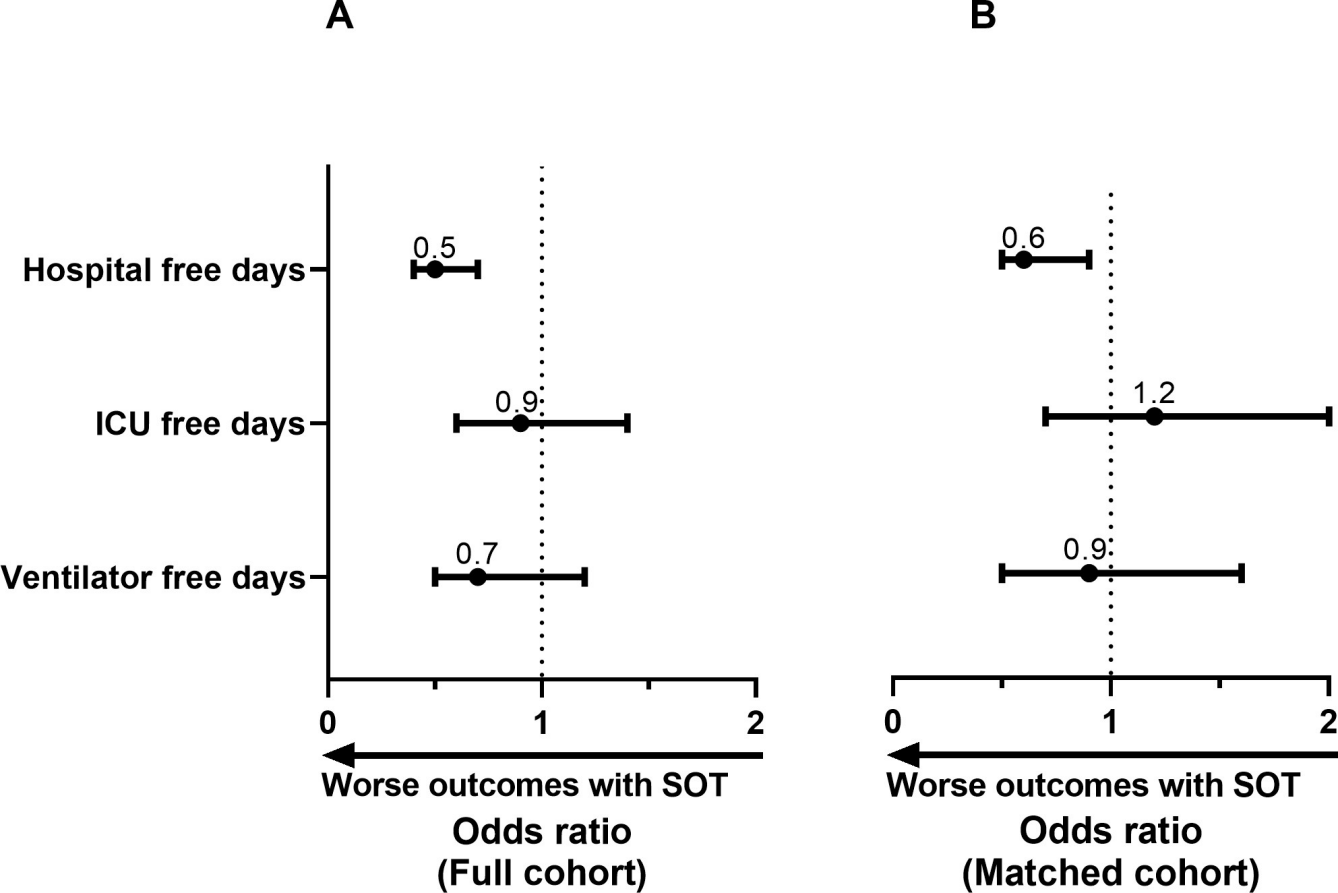
Odds ratio for hospital free days, ICU free days, and ventilator free days. ICU, intensive care unit; SOT, solid organ transplant; An odds ratio < 1 indicates worse clinical outcomes for SOT recipients compared to controls. Odds ratios were calculated using proportional ordinal regression.

As the primary analysis in the matched cohort, median HFDs were 17 for the SOT group and 21 for the control group (OR = 0.64, 95%CI 0.46 to 0.90, P = 0.01; S2 Table and Fig 1). Patients in the SOT group had fewer HFDs (increased healthcare utilization) than patients in the control group.

### 3.4. Secondary Outcomes: IFDs, VFDs, 28-day mortality, and 60-day mortality

**Fig 2:**
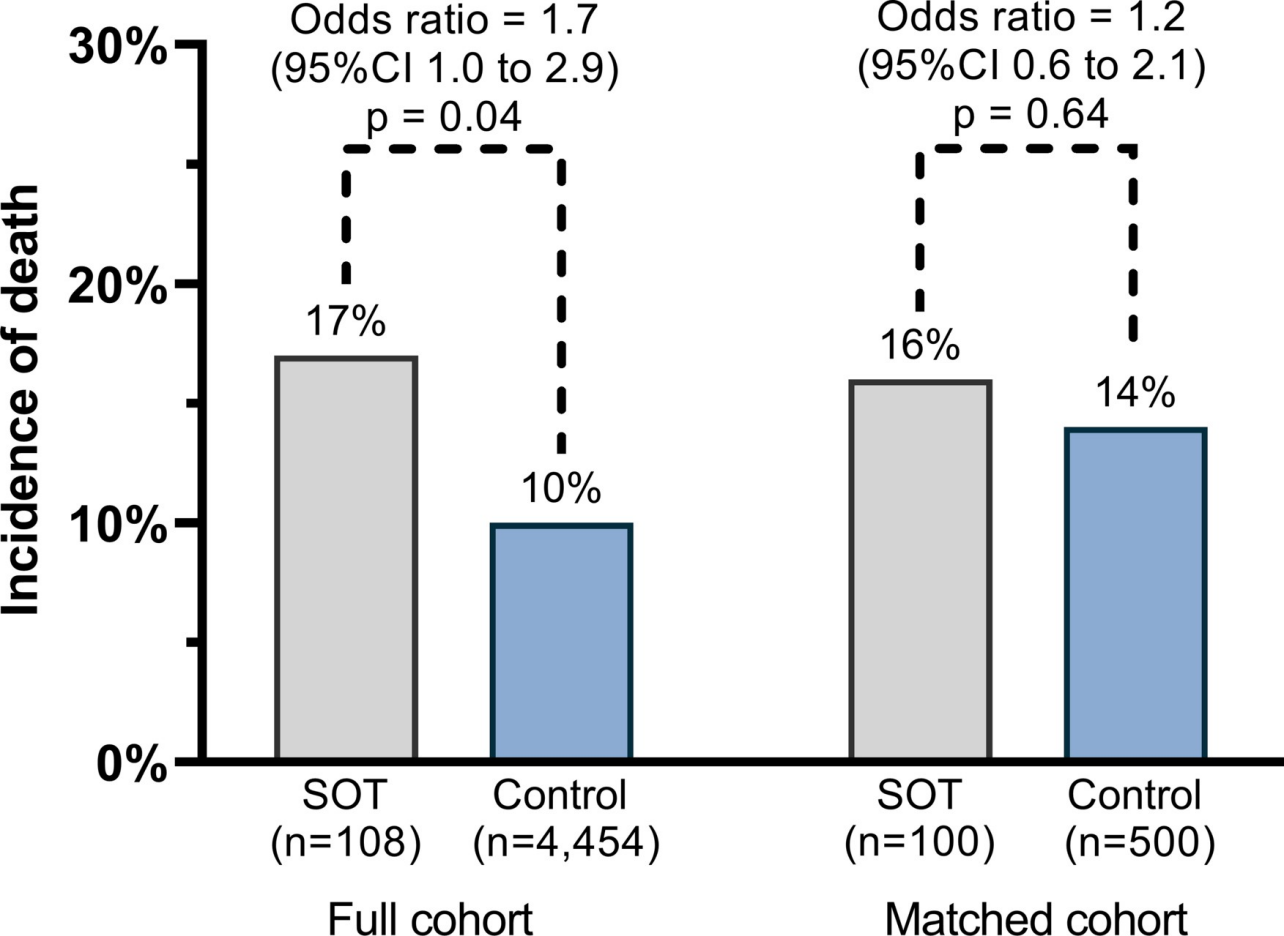
Incidence of 60-day mortality. SOT, solid organ transplant; The odds for 60-day mortality were statistically increased for SOT recipients in the full cohort. However, this association was attenuated in the matched cohort.

In the matched cohort, median IFDs were 28 for the SOT group and 28 for the control group (OR = 1.20, 95%CI 0.72 to 2.00, P = 0.49) and median VFDs were 28 for the SOT group and 28 for the control group (OR = 0.91, 95%CI 0.53 to 1.57, P = 0.75; S2 Table and Fig 1). The incidence of death at 28 days was 9% for the SOT group and 12% for the control group (OR = 0.76, 95%CI 0.36 to 1.57, P = 0.46). The incidence of death at 60 days was 16% for the SOT group and 14% for the control group (OR = 1.15, 95%CI 0.64 to 2.08, P = 0.64; Fig 2). We observed a larger number of delayed deaths (days 29 to 60) in the SOT group (7% vs 3%) and conducted a post-hoc analysis that identified increased odds for delayed death in the SOT group (OR = 2.96, 95%CI 1.11 to 7.90, P = 0.03).

### 3.5. Sensitivity analyses

Collapsing outcome data into 2-day increments satisfied the proportional hazards assumption. Results of these sensitivity analyses supported the findings of the primary and secondary analyses, whereby SOT was associated with fewer HFDs (OR = 0.65, 95%CI 0.46 to 0.92, P = 0.02) but not IFDs (OR = 1.09, 95%CI 0.66 to 1.78, P = 0.74) or VFDs (OR = 0.93, 95%CI 0.53 to 1.62, P = 0.80) in the matched cohort (S2 Fig). Using generalized estimating equations, SOT recipients had fewer HFDs (mean difference = -1.97 days, 95%CI -3.91 to -0.03, P = 0.047) but had similar IFDs (mean difference = -0.15 days, 95%CI -1.90 to 1.61, P = 0.87) and VFDs (mean difference = -0.32 days, 95%CI -1.95 to 1.30, P = 0.70) compared to control patients in the matched cohort analysis.

Prevalence of critical illness on admission was lower for SOT recipients in the propensity matched cohort (9% SOT group vs 16% control group). In a sensitivity analysis that adjusted for critical illness on admission in the matched cohort, SOT was associated with fewer HFDs (OR = 0.57, 95%CI 0.40 to 0.81, P = 0.001) but not IFDs (OR = 0.75, 95%CI 0.45 to 1.23, P = 0.25), VFDs (OR = 0.72, 95%CI 0.42 to 1.24, P = 0.24), 28-day mortality (OR = 0.90, 95%CI 0.41 to 1.94, P = 0.78), or 60-day mortality (OR = 1.28, 95%CI 0.68 to 2.40, P = 0.44).

### 3.6. Subgroup analyses on recent transplant and organ type

When stratified by year of most recent transplant as the 2019–2020 subgroup (24 SOT recipients with 120 matched controls) versus the ‘before 2019’ subgroup (84 SOT recipients with 420 matched controls), association with HFD retained statistical significance for the ‘before 2019’ subgroup. Although the associations suggested worse clinical outcomes for HFD and VFDs for the 2019–2020 subgroup, these were not statistically significant—potentially due to the small size of this subgroup (S3 Fig). When outcomes were stratified by organ type, no statistically significant associations were observed—likely due to small sizes of each subgroup (S3 Table). Although statistical significance was not observed, patients with heart or lung transplant may have fewer HFDs (increased healthcare utilization) and increased risk of 60-day death.

## 4. Discussion

This multihospital study evaluated clinical outcomes for 100 hospitalized SOT recipients and 500 propensity-matched control patients. Study groups were well-balanced on 10 meaningful covariates. In this propensity-matched cohort study, SOT recipients had fewer hospital-free days over a 28-day period, indicating increased risk for death or prolonged hospitalization compared to the control group. These results confirmed the study hypothesis. However, differences in secondary outcomes of 28-day ICU-free days, 28-day ventilator-free days, and 60-day mortality were not statistically significant.

In the full cohort, SOT recipients had an increased risk for 60-day mortality. However, SOT recipients had more chronic comorbidities. After adjusting for chronic comorbidities in the matched cohort, no difference in mortality was observed indicating that immunosuppression for SOT may not negatively impact health outcomes following hospitalization for COVID-19.

Our study builds on other studies that evaluated outcomes from COVID-19 among hospitalized SOT recipients [9–13] with important methodologic advances to account for inter-hospital patient transfer, evaluate healthcare utilization outcomes that account for death, and observe death up to 60 days. We evaluated outcomes across multiple hospital encounters across the health system due to inter-hospital COVID-19 patient transfer for changes in level of care and sequestering of COVID-19 patients into highly infectious disease units. The outcomes of HFD, VFD, and IFD evaluate healthcare resource utilization while simultaneously accounting for the competing event of death. Death was evaluated for up to 60 days to account for prolonged illness and organ injury. The 60-day death observation period appears to be a relevant study design feature as we identified a potential increased risk of delayed deaths (days 29–60) among SOT recipients. Future studies that evaluate COVID-19 outcomes among SOT recipients should consider observing death for at least 60 days following hospital admission.

Mortality reported in our study should be interpreted in the context of other published studies. Miarons et al. reported a non-statistically significant increase in 28-day mortality among 46 hospitalized SOT recipients compared to 166 hospitalized controls, who were matched on sex, age, and age-adjusted Charlson’s Index (37% vs 23%) [10]. Molnar et al. reported no difference in 28-day mortality among 98 critically ill SOT recipients compared to 288 critically ill controls, who were matched using a propensity score generated from many demographic variables (40% vs 43%) [11]. Avery et al. reported no difference in in-hospital mortality among 45 hospitalized SOT recipients compared to 2,427 hospitalized controls using logistic regression that was adjusted for severity score on admission (4% vs 11%) [9]. Linares et al. reported no difference in inpatient mortality among 41 hospitalized SOT recipients compared to 220 hospitalized controls (12% vs 15%), who were matched using a propensity score [12]. Rinaldi et al. reported no difference in 30-day mortality among 24 hospitalized SOT recipients compared to 861 hospitalized controls using multivariable regression (19% vs 22%) [13]. The 28-day mortality among hospitalized SOT recipients (9%) and propensity score matched controls (12%) in our study falls on the lower end of 28/30-day mortality reported for hospitalized SOT recipients (range, 6% to 37%) and controls (range, 10% to 23%) [9, 10, 13].

Even if health outcomes of hospitalized patients are similar, the COVID-19 pandemic may disproportionally impact SOT recipients’ risk for hospitalization. Of 12,084 patients who tested positive for COVID-19 at our health system from Mar to Jul 2020, 3,536 (29%) were hospitalized [20]. In comparison, 67% of a subgroup of SOT recipients with COVID-19 at our health system were hospitalized [5]. This is consistent with a hospitalization of 66% reported in a multicenter registry of SOT recipients with COVID-19 [21]. A large multicenter study reported that SOT recipients diagnosed with COVID-19 have an increased risk for hospitalization (RR = 3.38, 95%CI 3.18 to 3.60) compared to nontransplant patients in a crude analysis, and this risk remained statistically significant after propensity score matching (RR = 1.22, 95%CI 1.11 to 1.34) [22]. It is unclear if this increased risk of hospitalization is driven by differences in early clinical progression, increased surveillance by the transplant center, or both.

Similar to other health systems, SOT recipients in our study had a lower level of acuity on admission compared to non-SOT recipients [9, 13]. This suggests that the increased hospitalization may be driven by increased transplant center engagement/surveillance or a lower threshold of clinical criteria used for admission due to awareness that immunosuppression may increase risk for poor outcomes. After adjusting for critical illness on admission in our study, SOT recipients had fewer HFDs but similar IFDs, VFDs, 28-day mortality, and 60-day mortality compared to non-SOT recipients. Future research should aim to optimize clinical criteria used to admit SOT recipients with COVID-19, especially among emergency departments with limited experience caring for SOT recipients, as a strategy to optimize clinical outcomes.

### 4.1. Limitations

Medication and non-medication COVID-19 management strategies were not systematically collected for non-SOT recipients; therefore, evaluation of COVID-19 management strategies was not possible. This study carefully extracted information from the hospitals’ electronic health record and unstructured progress notes from transplant center coordinators to capture deaths that occurred after hospital discharge. However, deaths after hospital discharge may be underreported in control patients if the deaths occurred at other health systems or were not recorded our health system’s medical record. Multiple COVID-19 treatments and vaccines that are currently available to prevent or shorten hospitalization were not available in 2020 when study patients were diagnosed with COVID-19. It is unclear if access to COVID-19 vaccination will amplify or attenuate disparities in health outcomes between SOT recipients versus nontransplant patients, as SOT recipients have an inferior immune response to COVID-19 vaccines [23, 24].

### 4.2. Conclusion

Among patients hospitalized with COVID-19, SOT recipients who were taking baseline immunosuppressant therapy had fewer hospital-free days over a 28-day period but had similar ICU-free days, ventilator-free days, 28-day mortality, and 60-day mortality compared to propensity-matched control patients in this cohort study from a multihospital health system. Hospitalized SOT recipients appear to need additional days of hospital care but can achieve short-term mortality outcomes that are similar to non-SOT recipients. Although an increased risk for delayed mortality (days 29–60) was observed among SOT recipients in a *post hoc* analysis, future studies are needed to evaluate death for at least 60 days following hospital admission.

## Supporting information

S1 Fig Patient inclusion flow chart. ADI, area deprivation index; BMI, body mass index; COVID-19, coronavirus disease 2019; PCR, polymerase chain reaction; SOT, solid organ transplant.
(DOCX)

S2 Fig Odds ratio for hospital free days, ICU free days, and ventilator free days: Sensitivity analysis using 2-day increments of outcome variables. ICU, intensive care unit; SOT, solid organ transplant; An odds ratio < 1 indicates worse clinical outcomes for SOT recipients compared to controls. Odds ratios were calculated using proportional ordinal regression. In this sensitivity analysis, the original ordinal outcome variable (range 0 to 28) was collapsed into an ordinal categorical variable with fewer groups to ensure that the proportional odds assumption was upheld.
(DOCX)

S3 Fig Odds ratio for hospital free days, ICU free days, and ventilator free days: Subgroup analysis by year of last transplant. ICU, intensive care unit; SOT, solid organ transplant.
(DOCX)

S1 Table Example calculations of hospital-free days. Current status: D, dead; NH, alive and not hospitalized; H, hospitalize; HFDs, hospital free days.
(DOCX)

S2 Table Outcomes. SOT, solid organ transplant; ICU, intensive care unit; Data are reported as median (interquartile range); * Reported over a 28-day period, with a scale of 0 to 28 days. A lower number indicates increased healthcare utilization that accounted for death.
(DOCX)

S3 Table Outcomes in the matched cohort stratified by organ type. SOT, solid organ transplant; CI, confidence interval; HFD, hospital free days; IFDs, intensive care unit free days; VFDs, ventilator free days.
(DOCX)

## Abbreviations

ADI: area deprivation index
BMI: body mass index
CKD: chronic kidney disease
CURATOR: COVID-19 Surveillance and Outcomes Registry
COVID-19: coronavirus disease 2019 (i.e., SARS-CoV-2 virus)
ICU: intensive care unit
IMU: intermediate care units
CI: confidence interval
ED: emergency department
HFDs: hospital free days
IFDs: ICU free days
OR: odds ratio
PCR: polymerase chain reaction
RR: relative risk
SOT: solid organ transplant
STD: diff standardized difference
VFDs: ventilator free days
WHO: World Health Organization
