# Supplementary material for: Hospitalization and survival of solid organ transplant recipients with coronavirus disease 2019: A propensity matched cohort study
Source: PLoS One. 2022 Dec 19;17(12):e0278781. doi: 10.1371/journal.pone.0278781 (PMC9762563; doi:10.1371/journal.pone.0278781)
Supplement: S1 Fig — ADI, area deprivation index; BMI, body mass index; COVID-19, coronavirus disease 2019; PCR, polymerase chain reaction; SOT, solid organ transplant. (DOCX) [file pone.0278781.s001.docx]

**S1 Fig.** Patient inclusion flow chart

**
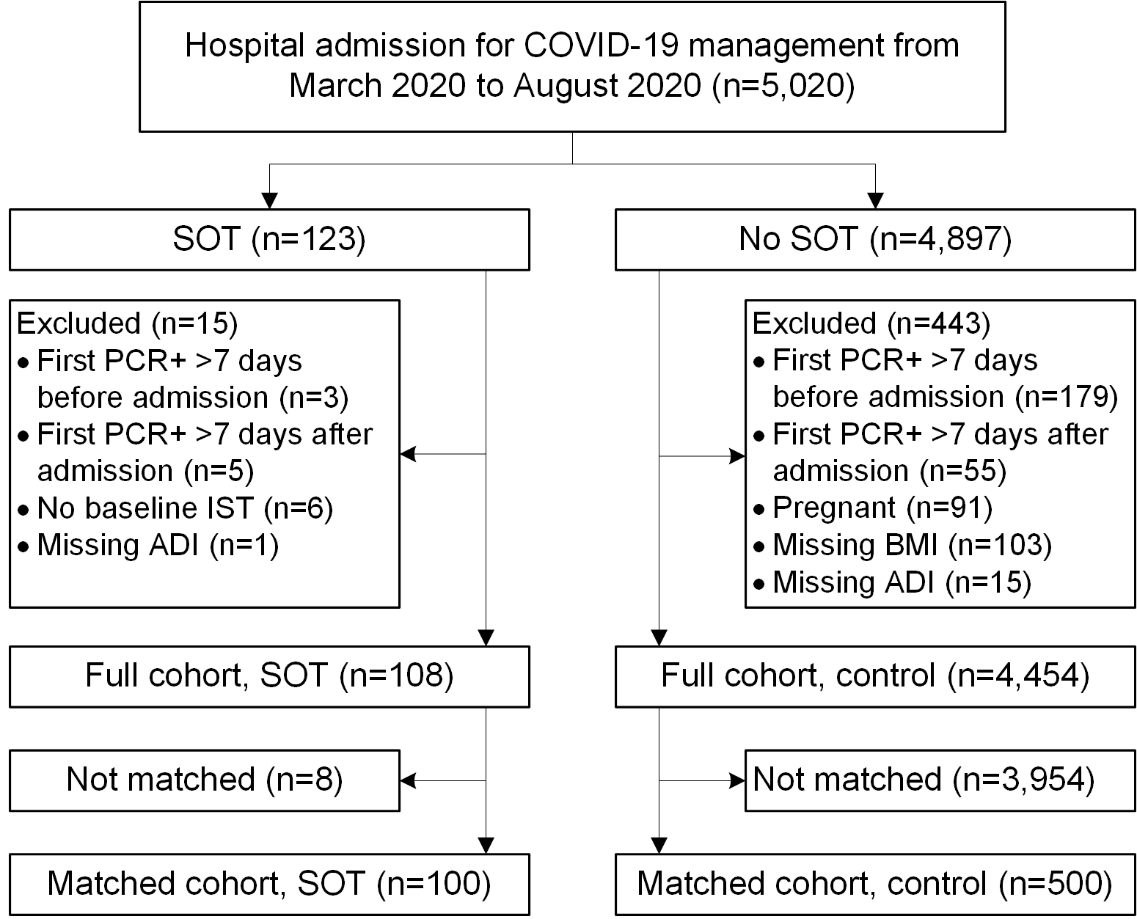
**

ADI, area deprivation index; BMI, body mass index; COVID-19, coronavirus disease 2019; PCR, polymerase chain reaction; SOT, solid organ transplant
