# Supplementary material for: Hospitalization and survival of solid organ transplant recipients with coronavirus disease 2019: A propensity matched cohort study
Source: PLoS One. 2022 Dec 19;17(12):e0278781. doi: 10.1371/journal.pone.0278781 (PMC9762563; doi:10.1371/journal.pone.0278781)
Supplement: S2 Fig — ICU, intensive care unit; SOT, solid organ transplant; An odds ratio < 1 indicates worse clinical outcomes for SOT recipients compared to controls. Odds ratios were calculated using proportional ordinal regression. In this sensitivity analysis, the original ordinal outcome variable (range 0 to 28) was collapsed into an ordinal categorical variable with fewer groups to ensure that the proportional odds assumption was upheld. (DOCX) [file pone.0278781.s002.docx]

**S2 Fig.** Odds ratio for hospital free days, ICU free days, and ventilator free days: sensitivity analysis using 2-day increments of outcome variables


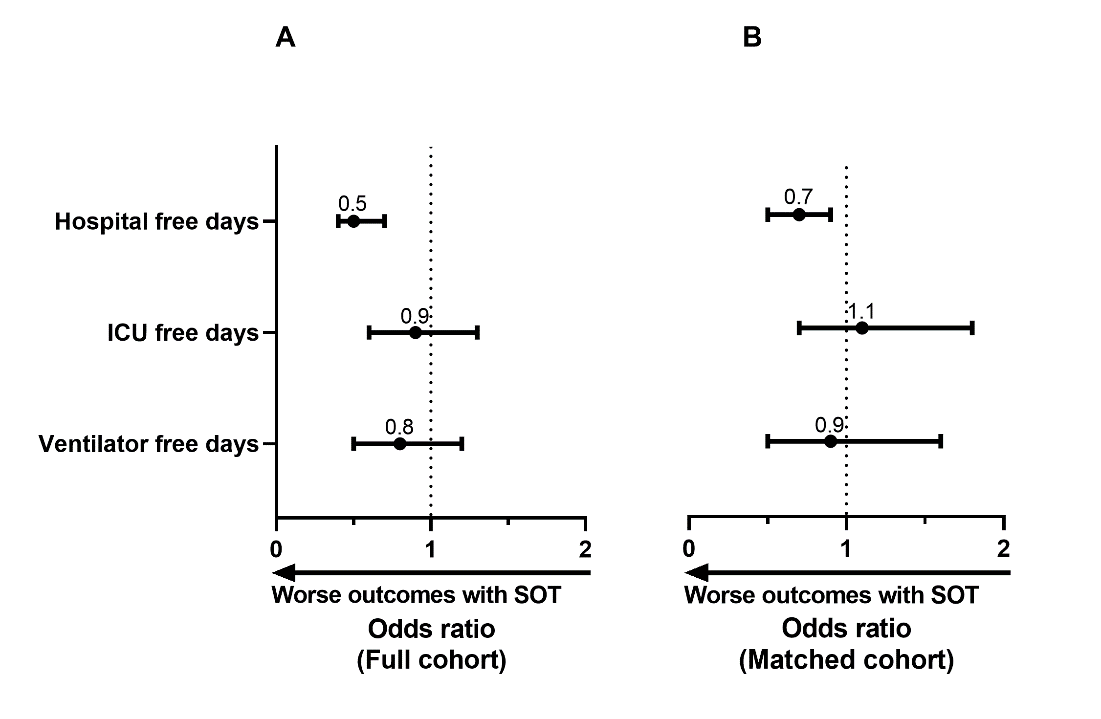


ICU, intensive care unit; SOT, solid organ transplant

An odds ratio < 1 indicates worse clinical outcomes for SOT recipients compared to controls. Odds ratios were calculated using proportional ordinal regression. In this sensitivity analysis, the original ordinal outcome variable (range 0 to 28) was collapsed into an ordinal categorical variable with fewer groups to ensure that the proportional odds assumption was upheld.
