# Supplementary material for: Hospitalization and survival of solid organ transplant recipients with coronavirus disease 2019: A propensity matched cohort study
Source: PLoS One. 2022 Dec 19;17(12):e0278781. doi: 10.1371/journal.pone.0278781 (PMC9762563; doi:10.1371/journal.pone.0278781)
Supplement: S3 Fig — ICU, intensive care unit; SOT, solid organ transplant. (DOCX) [file pone.0278781.s003.docx]

**S3 Fig.** Odds ratio for hospital free days, ICU free days, and ventilator free days: subgroup analysis by year of last transplant


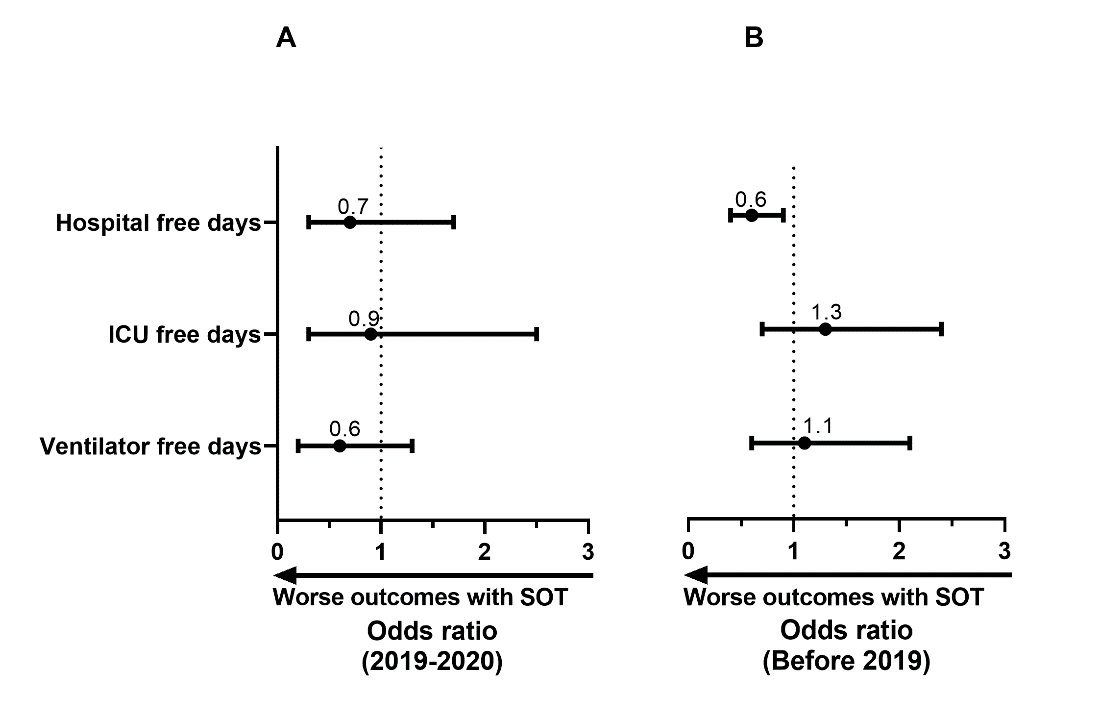


ICU, intensive care unit; SOT, solid organ transplant
