# Supplementary material for: Hospitalization and survival of solid organ transplant recipients with coronavirus disease 2019: A propensity matched cohort study
Source: PLoS One. 2022 Dec 19;17(12):e0278781. doi: 10.1371/journal.pone.0278781 (PMC9762563; doi:10.1371/journal.pone.0278781)
Supplement: S1 Table — Current status: D, dead; NH, alive and not hospitalized; H, hospitalize; HFDs, hospital free days. (DOCX) [file pone.0278781.s004.docx]

**S1 Table.** Example calculations of hospital-free days

| **Case** | **Day following hospital admission** | | | | | | | | | | | | | | | | | | | | | | | | | | | | **HFDs** |
| --- | --- | --- | --- | --- | --- | --- | --- | --- | --- | --- | --- | --- | --- | --- | --- | --- | --- | --- | --- | --- | --- | --- | --- | --- | --- | --- | --- | --- | --- |
|  | **1** | **2** | **3** | **4** | **5** | **6** | **7** | **8** | **9** | **10** | **11** | **12** | **13** | **14** | **15** | **16** | **17** | **18** | **19** | **20** | **21** | **22** | **23** | **24** | **25** | **26** | **27** | **28** |  |
| 1 | H | H | H | H | H | H | H | H | H | H | H | NH | NH | NH | NH | NH | NH | NH | NH | NH | NH | NH | NH | NH | NH | NH | NH | NH | 17 |
| 2 | H | H | H | H | H | NH | NH | NH | NH | H | H | H | H | H | H | H | NH | NH | NH | NH | NH | NH | NH | NH | NH | NH | NH | NH | 16 |
| 3 | H | H | H | H | H | NH | NH | NH | NH | H | H | H | H | H | H | H | **D** | **D** | **D** | **D** | **D** | **D** | **D** | **D** | **D** | **D** | **D** | **D** | 4 |
| 4 | H | H | H | H | H | H | H | H | H | H | H | H | H | H | H | H | H | H | H | **D** | **D** | **D** | **D** | **D** | **D** | **D** | **D** | **D** | 0 |
| 5 | H | H | H | H | H | H | H | H | H | H | H | H | H | H | H | H | H | H | H | H | H | H | H | H | H | H | H | H | 0 |

Current status: D, dead; NH, alive and not hospitalized; H, hospitalized

HFDs, hospital free days
