# Supplementary material for: Hospitalization and survival of solid organ transplant recipients with coronavirus disease 2019: A propensity matched cohort study
Source: PLoS One. 2022 Dec 19;17(12):e0278781. doi: 10.1371/journal.pone.0278781 (PMC9762563; doi:10.1371/journal.pone.0278781)
Supplement: S2 Table — SOT, solid organ transplant; ICU, intensive care unit; Data are reported as median (interquartile range); * Reported over a 28-day period, with a scale of 0 to 28 days. A lower number indicates increased healthcare utilization that accounted for death. (DOCX) [file pone.0278781.s005.docx]

**S2 Table.** Outcomes

|  | **Full cohort** | | **Matched cohort** | |
| --- | --- | --- | --- | --- |
|  | **SOT**  **(n=108)** | **Controls**  **(n=4,454)** | **SOT**  **(n=100)** | **Controls**  **(n=500)** |
| Hospital free days^*^ | 17 (8 – 23) | 22 (14 – 24) | 17 (8 – 23) | 21 (11 – 24) |
| ICU free days^*^ | 28 (26 – 28) | 28 (27 – 28) | 28 (26 – 28) | 28 (26 – 28) |
| Ventilator free days^*^ | 28 (28 – 28) | 28 (28 – 28) | 28 (28 – 28) | 28 (28 – 28) |

SOT, solid organ transplant; ICU, intensive care unit

Data are reported as median (interquartile range)

^*^ Reported over a 28-day period, with a scale of 0 to 28 days. A lower number indicates increased healthcare utilization that accounted for death.
