# Supplementary material for: Hospitalization and survival of solid organ transplant recipients with coronavirus disease 2019: A propensity matched cohort study
Source: PLoS One. 2022 Dec 19;17(12):e0278781. doi: 10.1371/journal.pone.0278781 (PMC9762563; doi:10.1371/journal.pone.0278781)
Supplement: S3 Table — SOT, solid organ transplant; CI, confidence interval; HFD, hospital free days; IFDs, intensive care unit free days; VFDs, ventilator free days. (DOCX) [file pone.0278781.s006.docx]

**S3 Table.** Outcomes in the matched cohort stratified by organ type

| Organ | SOT, n | Control, n | Odds ratio (95% CI) | | | 60-day death | | |
| --- | --- | --- | --- | --- | --- | --- | --- | --- |
|  |  |  | HFDs | IFDs | VFDs | SOT, % | Control, % | OR  (95% CI) |
| Kidney | 60 | 300 | 0.71  (0.45-1.13) | 1.37  (0.70-2.66) | 1.08  (0.51-2.29) | 15% | 15% | 1.00  (0.46-2.18) |
| Liver | 13 | 65 | 0.65  (0.25-1.66) | 0.92  (0.29-5.34) | 0.94  (0.26-3.44) | 8% | 12% | 0.59  (0.07-5.20) |
| Lung | 10 | 50 | 0.42  (0.12-1.40) | 0.46  (0.08-2.57) | 0.39  (0.06-2.42) | 20% | 14% | 1.57  (0.26-9.65) |
| Multiple | 12 | 60 | 0.73  (0.37-1.46) | 1.11  (0.33-3.75) | 0.92  (0.20-4.25) | 17% | 10% | 1.72  (0.33-9.07) |
| Heart | 5 | 25 | 0.36  (0.04-2.99) | 2.25  (0.09-54.0) | 0.86  (0.08-9.01) | 40% | 20% | 2.27  (0.36-14.21) |

SOT, solid organ transplant; CI, confidence interval; HFD, hospital free days; IFDs, intensive care unit free days; VFDs, ventilator free days
